# Using genetic variation for establishing causality of cardiovascular risk factors: overcoming confounding and reverse causality

**DOI:** 10.1007/s12471-014-0534-z

**Published:** 2014-03-04

**Authors:** R. A. J. Smit, S. Trompet, A. J. M. de Craen, J. W. Jukema

**Affiliations:** 1 Department of Gerontology and Geriatrics, Leiden University Medical Center, Albinusdreef 2, 2333 ZA Leiden, the Netherlands; 2 Department of Cardiology, Leiden University Medical Center, Albinusdreef 2, 2333 ZA Leiden, the Netherlands; 3 Interuniversity Cardiology Institute Netherlands, Utrecht, the Netherlands

**Keywords:** Cardiovascular risk assessment, Genetics, Instrumental variable analysis, Mendelian randomisation

## Abstract

Cardiovascular disease (CVD) remains the leading cause of death in developed countries, despite the decline of CVD mortality over the last two decades. From observational, predictive research, efforts have been made to find causal risk factors for CVD. However, in recent years, some of these findings have been shown to be mistaken. Possible explanations for the discrepant findings are confounding and reverse causation. Genetic epidemiology has tried to address these problems through the use of Mendelian randomisation. In this paper, we discuss the promise and limitations of using genetic variation for establishing causality of cardiovascular risk factors.

Cardiovascular disease (CVD) remains the leading cause of death in developed countries. This is unlikely to change within the near future, despite the decline of CVD mortality over the last two decades [1]. One of the pivotal studies that broadened our understanding of cardiovascular risk is the Framingham Heart Study. Since its inception in 1948, this study has identified various major risk factors contributing to CVD, including hypertension and elevated lipid concentrations [2]. Moreover, the Framingham Heart Study has generated one of the first multivariate cardiovascular risk prediction scores [3]. From observational, predictive research, efforts have been made to also assess likely causal relationships. However, in recent years, some of these findings have been called into question and ultimately proven wrong.

One of the most profound examples of such high-profile misidentification is the risk-lowering effect of hormone-replacement therapy on coronary heart disease found in observational studies, leading to widespread prescription of hormones for post-menopausal women. Subsequent randomised controlled trials (RCTs) showed that hormone therapy not only fails to lower cardiovascular risk, but may also even increase mortality risk and lead to other adverse clinical outcomes [4, 5]. Similar over-turnings were seen for vitamins E and C after RCTs disproved any cardioprotective effects [6]. It has been argued that the most likely explanations for these discrepancies have been confounding by environmental and behavioural factors, baseline health status, and prescription policies, combined with reverse causation and selection bias [7]. This shows that observational studies have certain weaknesses. Similar limitations might be present for RCTs, which are still viewed as the gold standard in estimation of causality. Firstly, it is sometimes unethical or impractical to allocate participants to exposures of interest (e.g. elevated blood pressure or physical inactivity). Additionally, participants are often relatively healthy with few co-morbidities which limits the applicability of the study findings to the general population, worsened by the possibility of consent bias. Lastly, trials may need significant follow-up time to produce meaningful results, which means RCTs are relatively resource-intensive and expensive.

Genetic epidemiology has tried to address these concerns through the use of Mendelian randomisation studies. While this term was introduced by Gray and Wheatley in 1991 [8], the underlying principles have long been recognised and applied in the field of econometrics, taking the form of instrumental variable analysis. An instrumental variable (or instrument) is a variable associated with the exposure, but not with the outcome of interest except through its association with the exposure [9]. The application of Mendelian randomisation in biomedical research, credited to Katan [10], is based on the concept that inheritance of germ line genetic variants is subject to the random allocation of alleles at conception, more commonly known as Mendel’s second law or the law of independent assortment [11]. As the associations between genotype and clinical outcome are generally unrelated to environmental or behavioural exposures, the use of single nucleotide polymorphisms (SNPs) known to be associated with modifiable risk factors makes it possible to avoid possible confounding or reverse causality (Fig. 1). In other words, causality of these risk factors can accurately be estimated using observational data in a research design resembling an RCT (Fig. 2) [12].

**Fig. 1:**
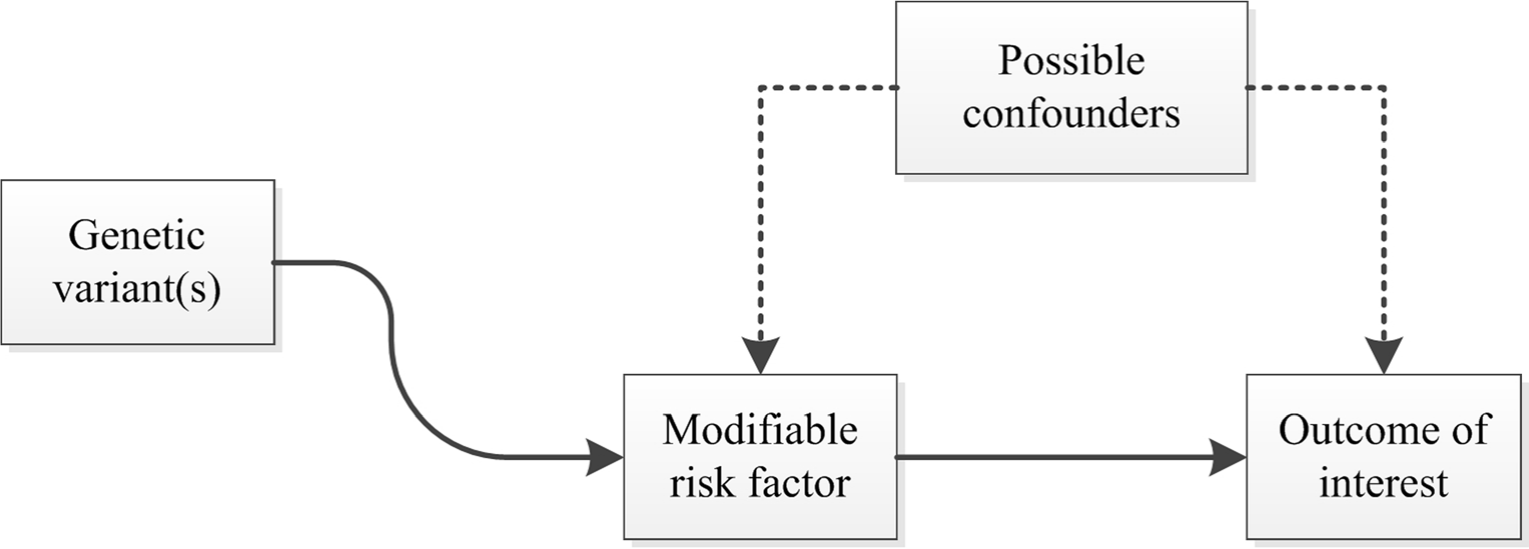
Causal relationships which satisfy the core assumptions of Mendelian randomisation: (1) genotype is associated with phenotype, (2) genotype is independent of confounding factors, and (3) genotype is associated with outcome, but only through phenotype

**Fig. 2:**
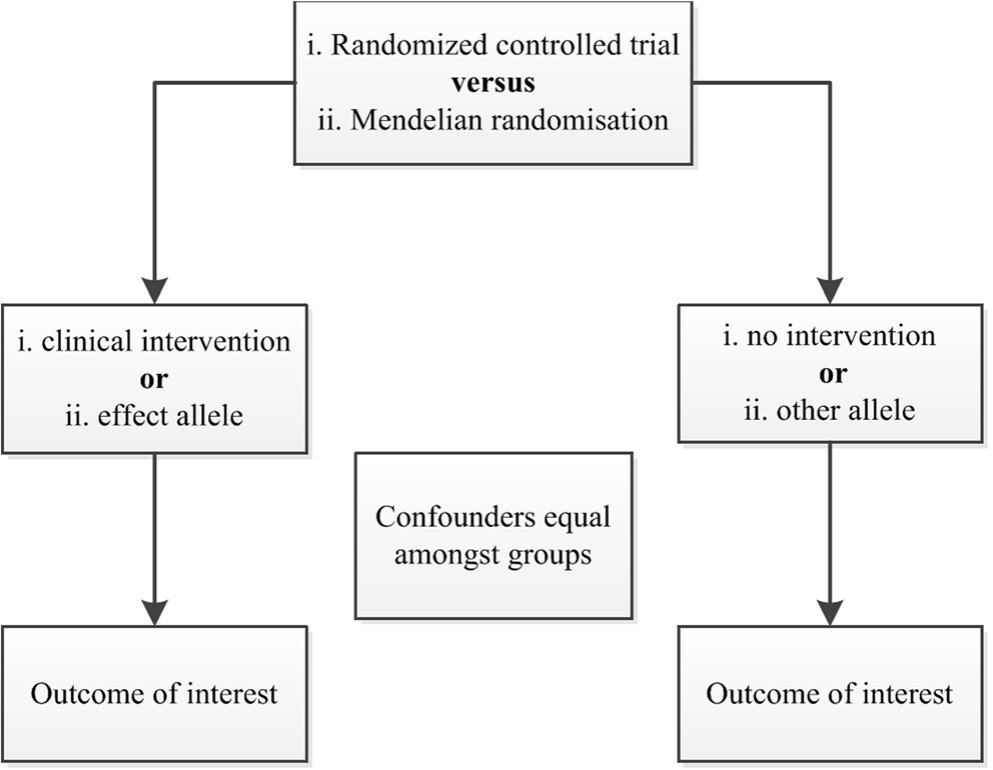
Comparison of randomised controlled trial and Mendelian randomisation study designs

A clear example where Mendelian randomisation was successfully used to prove the causality of a possible risk factor is the secretory phospholipase A2 (sPLA2) story. Higher circulating levels of sPLA2-IIA mass or sPLA2 enzyme activity have been associated with increased risk of cardiovascular events in observational studies [13]. However, a recent RCT with varespladib, a sPLA2 inhibitor, was stopped because of lack of efficacy. Subjects randomised to varespladib had an increased risk for cardiovascular events compared with subjects receiving placebo [14]. A Mendelian randomisation study was conducted to investigate the causality of sPLA2 in cardiovascular disease. The polymorphism rs11573156, which was associated with significantly lower sPLA2 levels, was not associated with coronary events (OR 1.02 (95 % CI 0.98–1.06)). The conclusion from this Mendelian randomisation study was that sPLA2-lowering therapy would not be a useful therapeutic tool to prevent cardiovascular disease [15].

To test whether elevated levels of C-reactive protein (CRP) are causally associated with ischaemic vascular disease, Zacho et al. conducted genotyping for four CRP polymorphisms. They found that the risk of ischaemic heart disease and ischaemic cerebrovascular disease was increased by a factor of 1.6 and 1.2, respectively, in persons who had CRP levels above 3 mg/l, as compared with persons who had CRP levels below 1 mg mg/l. Polymorphisms in the CRP gene were associated with considerable increases in CRP levels and thus with a theoretically predicted increase in the risk of ischaemic vascular disease. However, these polymorphisms were not associated with an increased risk of ischaemic vascular disease, thereby demonstrating that a causal relationship of CRP levels with adverse cardiovascular outcome is unlikely [16].

Another example of a Mendelian randomisation, originating from Katan’s original hypothesis, used apolipoprotein E (ApoE) genotype to infer causality between cholesterol and cancer [10, 17]. The background for this was the uncertainty whether the associations found between low plasma cholesterol levels and increased risk of cancer might actually reflect a hypocholesterolaemic effect of cancer in preclinical stages [10]. Trompet et al. reported that subjects within the lowest third of plasma cholesterol levels had increased risk of cancer incidence (HR 1.9 (95 % CI 1.34–2.70)) and cancer mortality (HR 2.03 (95 % CI 1.23–3.34)), when compared with those within the highest third of plasma cholesterol levels. However, they also found that carriers of the ApoE2 genotype, who had 9 % lower plasma cholesterol than carriers of the ApoE4 genotype, did not have increased risk of cancer incidence (HR 0.86 (95 % CI 0.50–1.47)) or cancer mortality (HR 0.70 (95 % CI 0.30–1.60)) compared with ApoE4 carriers. These findings suggested that low cholesterol levels are not causally related to increased cancer risk [17].

An important limitation of Mendelian randomisation is that genetic variants generally explain a modest amount of the variation in exposure levels, which means large sample sizes are needed to obtain valid results. It has been suggested that combining multiple SNPs into an allele score increases power and facilitates avoidance of weak instrument bias [18, 19]. Genome-wide association studies (GWAS), which scan large numbers of genetic markers in genomes of different individuals to find genetic variations associated with a particular disease or trait, have made construction of these genetic risk scores feasible. Teslovich et al. found 95 loci associated with plasma lipids in more than 100,000 individuals, explaining 9.6–12.4 % of total variance of lipid levels in the Framingham Heart Study and corresponding to ∼25–30 % of the genetic variance for each trait [20]. Other large-scale GWAS have examined traits of blood pressure [21], body mass index [22], and CRP [23], providing more insight into the genetics and biology of these possible risk factors.

Various studies have applied GWAS findings to examine causality of cardiovascular risk factors. For example, Voight et al. constructed a genetic risk score comprising 14 SNPs known to be associated with HDL cholesterol but not with other lipid traits. While observational epidemiology showed that an increase of 1 SD in HDL cholesterol was associated with decreased occurrence of myocardial infarction (OR 0.62 per SD (95 % CI 0.58–0.66)), genetically raised HDL was not associated with risk of myocardial infarction (OR 0.93 per SD (95 % CI 0.68–1.26)), thereby challenging the concept that raising plasma HDL cholesterol leads to reductions in risk of myocardial infarction. In contrast, the estimate from observational epidemiology for LDL cholesterol (OR 1.54 per SD (95 % CI 1.45–1.63)) was concordant with that from genetically raised LDL (OR 2.13 per SD (95 % CI 1.69–2.69)) [24]. In another recent study, a total of 30 SNPs were combined by Lieb et al. to evaluate whether hypertension truly acts as a causative factor for coronary artery disease, finding that those individuals carrying most systolic and diastolic blood pressure raising risk alleles had the highest odds of having coronary artery disease [25].

Most research has been performed using data from Caucasian populations only, which illustrates one of the limitations to the application of genetic risk scores in clinical practice. It is unlikely that Mendelian randomisation findings will uniformly translate into treatment effects as clinical interventions may have additional biological and biochemical pathways through which they affect clinical outcome, though the findings will generally be informative for the direction of effect and may further the design of an intervention study. In general, Mendelian randomisation studies must examine the possibility of potential confounders to genotype. This includes confounding through multiple functions of a genotype (pleiotropy), the non-random association of alleles at two or more loci (linkage disequilibrium), population stratification, and canalisation, which describes a foetal developmental change in response to a potentially harmful genetic variant [12].

Despite its current challenges, genetic epidemiology has great potential for extending the knowledge base of cardiovascular risk assessment. With increasing sample sizes and next-generation sequencing, GWAS will be able to detect increasing numbers of trait- and disease-associated genetic variants. Recently, the Global Lipids Genetics Consortium identified 157 loci associated with lipid levels, including 62 loci not previously associated with lipid levels in humans, thereby extending the findings of Teslovich et al. and opening up new possibilities for construction of genetic risk scores [26]. Moreover, in coming years, academic cooperation through international research consortia (e.g. CHARGE, GIANT, IDEAL) will present unprecedented possibilities for translational and (pre)clinical research.

## References

[1] Mendis S, Puska P, Norrving B, editors. Global atlas on cardiovascular disease prevention and control. Geneva: World Health Organization; 2011. Available from: www.who.int/cardiovascular_diseases/publications/atlas_cvd/en/.

[2] Kannel WB, Dawber TR, Kagan A (1961). Factors of risk in the development of coronary heart disease—six year follow-up experience. The Framingham Study. Ann Intern Med.

[3] Wilson PW, D’Agostino RB, Levy D (1998). Prediction of coronary heart disease using risk factor categories. Circulation.

[4] Rossouw JE, Anderson GL, Prentice RL (2002). Risks and benefits of estrogen plus progestin in healthy postmenopausal women: principal results from the Women’s Health Initiative randomized controlled trial. JAMA.

[5] Gabriel SR, Carmona L, Roque M, et al. Hormone replacement therapy for preventing cardiovascular disease in post-menopausal women. Cochrane Database Syst Rev. 2005;(2):CD002229. 10.1002/14651858.CD002229.pub2 PMC4164473 15846631

[6] Lawlor DA, Davey Smith G, Kundu D (2004). Those confounded vitamins: what can we learn from the differences between observational versus randomised trial evidence?. Lancet.

[7] Lawlor DA, Harbord RM, Sterne JA (2008). Mendelian randomization: using genes as instruments for making causal inferences in epidemiology. Stat Med.

[8] Gray R, Wheatley K (1991). How to avoid bias when comparing bone marrow transplantation with chemotherapy. Bone Marrow Transplant.

[9] Greenland S (2000). An introduction to instrumental variables for epidemiologists. Int J Epidemiol.

[10] Katan MB (1986). Apolipoprotein E, isoforms, serum cholesterol, and cancer. Lancet.

[11] Mendel G. Experiments in plant hybridization. 1865. Available from: www.mendelweb.org/archive/Mendel.Experiments.txt.

[12] Davey Smith G, Ebrahim S (2005). What can mendelian randomisation tell us about modifiable behavioural and environmental exposures?. BMJ.

[13] Koenig W, Khuseyinova N (2009). Lipoprotein-associated and secretory phospholipase A2 in cardiovascular disease: the epidemiological evidence. Cardiovasc Drugs Ther.

[14] Nicholls SJ, Kastelein JJ, Schwartz GG (2014). Varespladib and cardiovascular events in patients with an acute coronary syndrome: the VISTA-16 randomized clinical trial. JAMA.

[15] Holmes MV, Simon T, Exeter HJ (2013). Secretory phospholipase A(2)-IIA and cardiovascular disease: a Mendelian randomization study. J Am Coll Cardiol.

[16] Zacho J, Tybjaerg-Hansen A, Jensen JS (2008). Genetically elevated C-reactive protein and ischemic vascular disease. N Engl J Med.

[17] Trompet S, Jukema JW, Katan MB (2009). Apolipoprotein e genotype, plasma cholesterol, and cancer: a Mendelian randomization study. Am J Epidemiol.

[18] Pierce BL, Ahsan H, Vanderweele TJ (2011). Power and instrument strength requirements for Mendelian randomization studies using multiple genetic variants. Int J Epidemiol.

[19] Burgess S, Thompson SG (2013). Use of allele scores as instrumental variables for Mendelian randomization. Int J Epidemiol.

[20] Teslovich TM, Musunuru K, Smith AV (2010). Biological, clinical and population relevance of 95 loci for blood lipids. Nature.

[21] Ehret GB, Munroe PB, Rice KM (2011). Genetic variants in novel pathways influence blood pressure and cardiovascular disease risk. Nature.

[22] Speliotes EK, Willer CJ, Berndt SI (2010). Association analyses of 249,796 individuals reveal 18 new loci associated with body mass index. Nat Genet.

[23] Dehghan A, Dupuis J, Barbalic M (2011). Meta-analysis of genome-wide association studies in >80 000 subjects identifies multiple loci for C-reactive protein levels. Circulation.

[24] Voight BF, Peloso GM, Orho-Melander M (2012). Plasma HDL cholesterol and risk of myocardial infarction: a Mendelian randomisation study. Lancet.

[25] Lieb W, Jansen H, Loley C (2013). Genetic predisposition to higher blood pressure increases coronary artery disease risk. Hypertension.

[26] Willer CJ, Schmidt EM, Sengupta S (2013). Discovery and refinement of loci associated with lipid levels. Nat Genet.

